# The culture of *JACMP*, the sanctity of editorial independence, and why your ego is not your amigo

**DOI:** 10.1002/acm2.70368

**Published:** 2025-11-28

**Authors:** Michael D. Mills

**Affiliations:** ^1^ Department of Radiation Oncology University of Louisville Louisville Kentucky USA

The single biggest problem in communication is the illusion that it has taken place.

— George Bernard Shaw (who might have been a ferocious peer reviewer with *legendary* comment threads.)^1^

**Welcome Aboard the S.S. *JACMP* and Encounter the Culture**

Academic journals are ships. They float on oceans of words, are steered by a hackneyed editorial crew who spends a great deal of time debating about which way the winds of change are blowing. Inside the fleet of the AAPM, two main vessels stand out:

- ***Medical Physics***—a proud, baroque galleon, bristling with equations, simulations, and fealty to the Principality of Monte Carlo. It prizes theory, elegance, and peer reviews that resemble challenges to doctoral defense presentations at the University of Helm's Deep.^2^, ^3^
- ***The Journal of Applied Clinical Medical Physics* (*JACMP*)**—a lean, clinically‐driven frigate, all sails up, asking, “Yes, but does this missive help image the patient before treating the next fraction?” It also reveres Monte Carlo, but in a more pragmatic, working‐vessel kind of way.

*JACMP* was not spawned from the academic ether. It *evolved*, as many things do—like volcanos, river beds, or acerbity—from a global imperative: a place to *communicate* work that is practical, principled, and patient‐first.

***JACMP* in a Nutshell (Albeit, a Heavily Annotated One)**

- **Clinically Obsessed**—We esteem research that speaks fluent clinic. We are not allergic to theory, but we get jazzed when science meets *patient charts*.
- **Globally Inclusive**—Open access for everyone, everywhere. Whether you are abstracting in a research hospital in Madison, Wisconsin, or reviewing proofs at a clinic in São Paulo, Brazil, it is yours—no paywalls, no strings, no wallet, and soul‐sapping login screens.
- **Double Blind Peer Review**—Everyone dons the metaphorical blindfold. Not because we are playing pin‐the‐Flaw‐on‐the‐Methods Section, but because Fairness *Matters*. Also, it prevents the “Did I not just Torpedo this dude's last manuscript?” syndrome.
- **Radically Real‐World**—QA? Yes. Workflow improvements? Yes. Training, safety, and clinical implementation? *Double yes!* If it helps someone practice safer, smarter, kinder physics—you are our kind of people.
- **Author Respect as a Core Value**—Publishing with us costs $900, your nights, too much of your family time, and probably your lunch breaks. **You often get *NO* credit from your employer for publishing**. The least we can do is not treat peer review like an audition for *Braveheart* while armed with a period faithful mace.
- **Visibility Equals Impact**—Open access papers get downloaded and cited more—sometimes triple. Turns out, people actually like reading stuff they do not have to *open a vein* to access; this decodes to more views and citations, and maybe an office with a window.
- **We are Not Just Growing—We are Post‐Puberty‐Level Stubborn**—400+ articles projected in 2025. We are publishing more, expanding our publication space, reaching farther, and yes, our impact factor is starting to demand its own coffee mug. *Nary a plateau in site*.

We have been planning this since 1997, which makes us legally old enough to rent a Mini Cooper S, adopt a leopard gecko, and insist on editorial autonomy like a seasoned, marginally grumpy uncle at Thanksgiving.

Management is doing things right; ***leadership is doing the right things***. (emphasis mine).

— Peter Drucker^4^

**Editorial Independence: It is Not Just a Polysyllabic Vibe**

**Ann Michael**: Editorial independence at its core means that a publisher may never tell an Editor in Chief (EIC) what he or she can or cannot publish in the journal. The EIC is the arbiter of what content is suitable for the journal, based on its mission and goals. For article, accept/reject decisions this is relatively straightforward.

The publisher also has a remit. The publisher is charged with the financial health of the journal. Sales and licensing, marketing, branding, distribution, and new product development are often the purview of the publisher. But the lines are never crystal clear. For example, new product development often requires the collaboration of publishing and editorial to define a product or service that is both viable in the market and valuable to the user community.

— The Scholarly Kitchen—Ask the Chefs: What is Editorial Independence and How Does It Impact Publishing? ^5^

While some of our papers are very fashionable with a plethora of citations and downloads, the *JACMP* is not here to impress university ranking committees or engage in academic robot wars. Our mission is precise: **serve clinical physicists and the patients they attend**. And to do that well, editors need *autonomy*—not a micromanaging boardroom, not 3 a.m. obsessive‐emails, and *definitely* not political horse‐trading.

This is not a quaint editorial request—it is *codified ethics*, per COPE,^6^ ICMJE,^7^ and AAPM's own policy scripture (see AP‐139‐A^8^).

**What To Do**:

- Appoint editors who are *excellent and experienced*, not just compliant.
- Let them publish what needs to be said, even if it makes you squirm a little.
- Understand that trust in a journal is *fragile*. Once broken, it is about as easy to rebuild as a stellar reputation on Retraction Watch.

**What *Not* to Do**:

- Do not push an article just because it flatters or serves the interests of leadership. That is not peer review; that is, marketing.
- Do not panic when the journal publishes something constructively critical. It means we are doing our job.
- Do not confuse “budget oversight” with “editorial veto power.” This is not *Monopoly* and journal authors do not live in hotels on *Boardwalk*.
- Do not cheat the system. If you long to pay association bills on the backs of your Journals, consider: COPE, ICMJE and the AAPM recommend Editors‐in‐Chief make all final publication decisions and are to be shielded from divergent society opinions and financial expectations.

**When Editorial Independence is Compromised**:

- The mission becomes an afterthought.
- Authors find better homes.
- Editors vamoose.
- Reviewers vanish like Schrödinger's cat^9^—neither alive nor dead (nor responding).
- And the journal becomes a cautionary tale whispered in conference vestibules.
  Your ego is writing checks your manuscript can't cash.
  — Not Top Gun (but maybe celebrity reviewer #2 commenting before their morning coffee.)^10^

**Your Ego Is Not Your Amigo (And Might Be Desk Rejected)**

Ego is the invisible saboteur of otherwise publishable work. It creeps in with:

- Claims that oversell the results.
- Limitations that vanish mysteriously in revision.
- Citations that avoid everyone else's work (unless it is your own).
- Author–reviewer crossfire that reads like courtroom transcripts over a family will.
- Reviewer comments that confuse constructive critique with a *Pattonesque monologue*.^11^

Peer review is not *Mortal Kombat*.^12^ It is collaborative inventiveness and serves a purpose: It is more like karaoke or modern dance—you are supposed to dance with the reviewer and sing your way to a better paper, so do it with some rhythm and refinement. Like Gene Kelly: Remember, **“Singing *in the Rain*** ^13^ ***”*** is the key to benefiting our patients and may be helpful for advancing your career.

The best authors know:

- No journal owes you publication. No, not even you.
- ***Good*** science can survive and thrive with stern and professional criticism.
- Taking feedback graciously is a hallmark of professional respect.
- That true prestige is in **serving the community**, not chasing high JIF trophy pubs like *Rocky* (Balboa) chases chickens.^14^
  “All scientists agree—when you censor the ones who don't.”
  — Jacobus MacFarlane (and perhaps that one post‐doc who makes everyone slightly nervous but is *usually* right.)^15^

**Some Parting Thoughts From a Departing Editor**

I have seen the *JACMP* rise from little more than a *Man of La Mancha* impossible dream to a trusted, international voice in clinical physics. As I hand over the reins, I have this admonition:

**Respect the *codified ethics*, Protect *journal independence* and Preserve the *JACMP culture***

We do not publish just for prestige—we publish with purpose, curiosity, and a whole lot of collaborative audacity. Let us keep doing that.

History is not only written by the Great People. Sometimes it is rewritten by visionary clinical physicists, idealists who venerate open access, and after midnight authors who take risks and change the world.

— Possibly you, next time you hit “Submit”. ^16^

## AUTHOR CONTRIBUTIONS

Michael Mills was responsible for conceptualization, content, investigation, writing, reviewing, and editing.

## CONFLICT OF INTEREST STATEMENT

The author reports no relevant conflicts of interest related to this work. The author is the editor‐in‐chief for the *JACMP* and is compensated by the AAPM for this service. ChatGPT 5.0 was used in the grammar review and proofreading of this editorial.
